# Challenges, innovations and considerations for the use of tongue swabs in *Mycobacterium tuberculosis* complex detection

**DOI:** 10.1371/journal.pone.0325585

**Published:** 2025-10-27

**Authors:** Anura David, Keneilwe Peloakgosi-Shikwambani, Zanele Nsingwane, Violet Molepo, Wendy Stevens, Lesley Scott

**Affiliations:** 1 Wits Diagnostics Innovation Hub, Health Sciences Research Office, Faculty of Health Sciences, University of the Witwatersrand, Johannesburg, South Africa; 2 National Priority Programmes, National Health Laboratory Services, Johannesburg, South Africa; St Petersburg Pasteur Institute, RUSSIAN FEDERATION

## Abstract

**Background:**

Sputum-based testing is most used for diagnosing pulmonary tuberculosis (TB), but non-sputum specimens such as tongue swabs (TS) offer additional approaches for detecting *Mycobacterium tuberculosis* complex (MTBC). This two-phase study evaluated various storage, transport, and processing conditions to improve MTBC detection using TS tested on quantitative PCR (qPCR) and Xpert MTB/RIF Ultra (Ultra).

**Methods:**

Adults (≥18 years) with Ultra-positive sputum were enrolled at a healthcare facility in Johannesburg, South Africa. In Phase 1, five serial TS were collected per participant and transported “dry” at 2–8°C, to the testing laboratory. In Phase 2, seven TS were collected: three stored “dry,” two in Tris-EDTA (TE) buffer, and two in PrimeStore® Molecular Transport Medium (MTM). Across both phases, pre-testing methods included heat lysis (HL)-only or HL combined with vortex bead beating (VBB), and storage at varying temperatures before qPCR. Swabs stored in MTM were tested on Ultra.

**Results:**

A paired *t*-test revealed no statistically significant differences in Ct values between sequentially collected TS (adjusted **p* *> 0.05). Similarly, mean IS*6110*/IS*1081* Ct values did not differ between fresh and frozen samples (t (85) = −0.031, *p* = 0.98; 95% CI: –0.44 to 0.43). VBB significantly reduced Ct values compared to HL alone (t (85) = −9.67, *p* = 2.422 x 10^-15^; 95% CI: −2.44 to −1.61). While storage conditions influenced Ct value and consistency to some extent, mean Ct values varied slightly from 32.48–34.28. Ultra detection improved when TS were processed with MTM and SR buffer.

**Conclusions:**

MTBC detection from serially collected TS was variable, but Ct values were consistent across swab order and storage conditions. VBB improved yield on qPCR, and Ultra detection was enhanced with diluted SR buffer, highlighting the value of optimized processing. These findings support the continued development of TS-based TB diagnostics.

## Introduction

The diagnosis of tuberculosis (TB) typically relies on sputum and other respiratory specimens for bacteriological confirmation [1]. However, additional specimen types such as urine [2], stool [3] and more recently tongue swabs (TS) [4] have shown value in detecting *Mycobacterium tuberculosis* complex (MTBC). Tongue swabs offer a non-invasive option for populations where sputum collection is difficult, such as children, people living with HIV (PLHIV), or individuals with paucibacillary disease [4]. Multiple studies have demonstrated the feasibility of detecting MTB DNA from tongue dorsum samples using molecular methods such as Xpert® MTB/RIF Ultra (Ultra) (Cepheid, Sunnyvale, CA, USA) [5,6]. Compared to sputum, TS are easier to collect, require minimal training and are acceptable to patients [7]. Swabs also have an added advantage in that they can be self-collected [7]. While sensitivity tends to be lower than that of sputum, especially in individuals with low bacterial loads [7] ongoing research is focused on improving sensitivity and facilitating implementation in resource-limited settings.

Since 2021, our group has been exploring the utility of TS for the detection of MTBC, at a time when limited published evidence existed to inform specimen collection, processing protocols, or performance expectations. As with any novel specimen type, evaluating TS for TB diagnosis requires rigorous research and protocol optimization to understand its operational and diagnostic potential. While studies using contrived samples play an important role in early-stage development, clinical validation in real-world settings is essential to assess true diagnostic utility. While Ultra has shown promise for use on TS, it represents just one option for testing. Furthermore, Ultra’s cartridge-based format may present limitations for high-volume screening while quantitative PCR (qPCR) offers a scalable, high-throughput alternative and was therefore investigated in this study.

This paper presents findings from an early two-phase study that investigated whether TS performance for MTBC detection is influenced by storage and transport conditions, including temperature and buffer preservation compared to dry transport and storage. Additionally, the study assessed the impact of laboratory processing modifications on detection yield.

## Materials and methods

### Ethics approval and consent to participate

Ethics approval for this study was obtained from the University of the Witwatersrand Human Research Ethics Committee (M1911150). All participants provided written informed consent.

### Study design and TS collection

In this cross-sectional study, we recruited adult (≥ 18 years) participants with Ultra-positive sputum results from the Hillbrow Community Health Centre in Johannesburg, South Africa. Participants were approached and invited to enroll in the study. Recruitment was performed over two phases. In phase 1, five serially collected TS were collected by a research nurse and in phase 2, seven serially collected TS were collected, after obtaining written informed consent. For both phases, TS were collected when participants returned to the healthcare facility to receive their Ultra results, prior to the initiation of TB treatment. Swabs were pre-numbered from 1 to 5 (or 1–7), but instead of collecting them sequentially by number, swabs were randomly selected without regard to their assigned number for collection. TS collection followed the method described by Andama *et al.* [8] with TS collected approximately two minutes apart. Each TS was stored in a 2 mL cryovial and transported to the research laboratory in Braamfontein, Johannesburg, for testing.

#### Phase one.

This phase had two primary objectives: first, to assess whether assay performance differed between TS tested within 24 hours of collection and those stored at −80°C prior to testing; and second, to evaluate whether vortex bead beating (VBB) improved MTBC detection compared to heat lysis (HL) alone, when tested on quantitative polymerase chain reaction (qPCR). Participant recruitment took place from 19 October 2022–15 March 2023. Thirty-eight participants provided consent. All five TS were transported “dry” or without any buffer, at 2–8°C, to the laboratory.

#### Phase two.

Findings from Phase 1 informed the design of Phase 2, where the primary objectives were to evaluate whether TS performance could be enhanced through alternative transport and storage conditions, and to determine whether refrigeration is necessary prior to laboratory processing. In addition, we assessed whether there were any differences in MTBC detection across the TS. Between 18 May and 21 August 2023, an additional 39 participants were enrolled, with seven TS serially collected from each. Three TS were transported ‘dry’, two in Tris-EDTA (TE) buffer (10 mM Tris-HCl, 1 mM EDTA Na₂, pH 8.0; Merck, Johannesburg, South Africa), and two in PrimeStore® Molecular Transport Medium (MTM; Longhorn Vaccines & Diagnostics, Bethesda, MD, USA). All TS were transported to the laboratory at 2–8°C, except those in MTM, which were transported at room temperature in either 1.5 mL or 2.2 mL volumes. Phase 2 also assessed two additional processing protocols for Ultra: MTM alone, and MTM combined with SR buffer. It was hypothesized that SR buffer might be necessary for optimal assay performance; however, because TS do not require sputum liquefaction, concerns were raised that undiluted SR could be too harsh. As such, a diluted SR buffer was evaluated to determine its suitability for TS processing.

### Tongue swab processing at the laboratory

#### Phase one.

**Fig 1:**
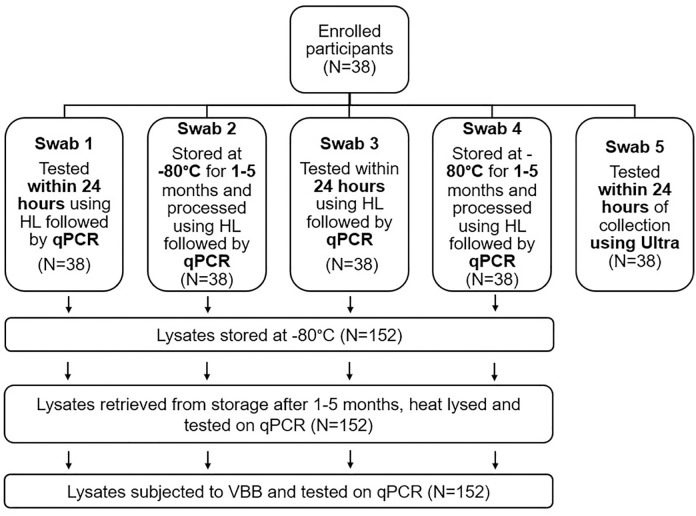
Schematic of tongue swab collection and testing strategy for participants enrolled in Phase 1 (n = 38). TS 1, 3, and 5 were tested within 24 hours of collection using either quantitative PCR (qPCR) or Xpert MTB/RIF Ultra (Ultra). TS 2 and 4 were stored at −80°C for 1–5 months prior to testing by qPCR. All TS processed by qPCR were initially subjected to heat lysis (HL) only, and the lysates were stored; stored lysates were then subjected to an additional HL step followed by vortex bead beating (VBB).

Upon receipt at the laboratory, TS were processed as described in Fig 1.

For pre-processing of the fresh and frozen TS (TS 1–4), 400 µl of TE buffer was added to each cryovial containing the TS, and lysed in a heating block for 10 minutes at 95°C. The lysate was then used as a template on a real-time qPCR targeting the IS*6110* and IS*1081* genes, using primer and probe design from the Quantigen group [9] with testing performed on the QuantStudio 5 system (Thermo Fisher Scientific, Waltham, MA, USA). Thresholds of 0.06 and 0.05 were used for the IS*6110* and IS*1081* genes, respectively to standardize cycle threshold (Ct) values. If only one MTBC target amplified with a Ct ≥ 38, the test was interpreted as inconclusive and repeated. Lysates were frozen at −80°C for 1–5 months after which they were retrieved from storage and thawed. HL was repeated, followed by qPCR. The heat lysed TS were then subjected to 5-minute VBB using a vortex and bead beating attachment (SI-H524, Horizontal Microtube Holder (24 tubes), Scientific Industries Inc, NY, USA). Bead beaten lysates were also tested on qPCR.

For TS Ultra testing, 2.2 mL Sample Reagent (SR) (Cepheid, Sunnyvale, CA, USA) was added to the TS, incubated at room temperature for 15 minutes and 2mL of the specimen was added to the Ultra cartridge and tested as per manufacturer instructions.

#### Phase two.

**Fig 2:**
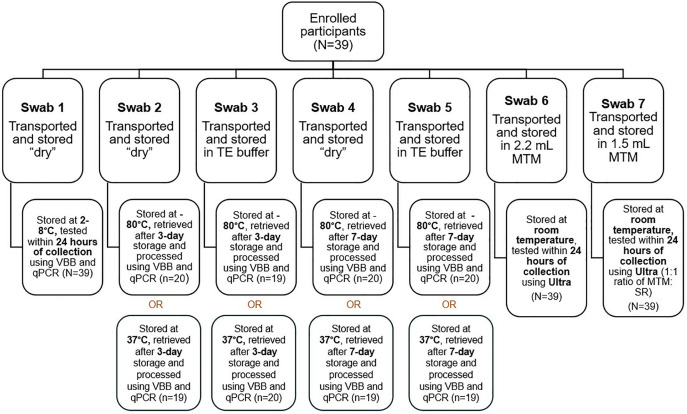
Overview of tongue swab (TS) collection, transport, storage conditions, and testing methods for participants in Phase 2 (n = 39). Tongue swabs were stored “dry,” in Tris-EDTA (TE) buffer, or PrimeStore® MTM, and tested using qPCR or Ultra after various storage durations (24 hours to 7 days) and temperatures (2–8°C, −80°C, 37°C, or room temperature). All TS tested on qPCR were subjected to a 10-minute HL before VBB.

Laboratory results from phase one which showed that VBB improved detection of MTBC, compared to HL alone, guided laboratory testing for phase two. One TS was tested within 24 hours of collection, by performing VBB followed by qPCR. Two TS were stored at −80°C and another two at 37°C. After a 3-day and 7-day incubation, one TS from each storage temperature was retrieved and tested using VBB and qPCR (Fig 2). As per the phase 1 protocol, 400 µl of TE buffer was added to all “dry” TS. For biosafety consideration and to assist with the lysis of the Mycobacteria, all TS tested on qPCR were subjected to a 10-minute HL before VBB.

For TS Ultra testing, 2mL MTM was removed from the vial containing 2.2 mL MTM and added directly to the Ultra cartridge. For the TS containing 1.5 mL MTM, 1.5 mL SR was added, incubated for 15 minutes at room temperature and 2mL used for Ultra testing. Once the specimen was added to the Ultra cartridge, testing was performed as per manufacturer instructions.

#### Statistical analysis.

In phase 1, to evaluate whether the storage condition (fresh vs −80°C) affected the average Ct values for the IS*1081* target, we performed a paired *t*-test. This test assessed the null hypothesis that there is no difference in mean Ct values between the two groups. Statistical analyses were conducted using R version 4.3.3, with a significance threshold of α = 0.05.

To compare the mean Ct values between two different extraction methods, HL and VBB, in phase 1, a paired *t*-test was conducted. This test was chosen as specimens were collected from the same individual. The null hypothesis tested whether there was no difference in mean Ct values between the two pre-processing methods.

In phase 2, to evaluate whether sequentially collected TS from the same participant produced significantly different Ct values, a one-way ANOVA was performed. Swab number was used as the independent variable, and Ct values were treated as the dependent variable. Observations with missing Ct values (n = 211) were excluded from the analysis. The Benjamini-Hochberg (BH) procedure was applied to adjust p-values for multiple testing.

To evaluate the effect of storage conditions on Ct values, a three-way ANOVA was performed using temperature, “dry” and buffer storage, and storage period as independent variables, and Ct value as the dependent variable. Interaction effects between variables were also tested. Observations were grouped by condition: temperature (2°C to 8°C, 37°C, −80°C), buffer or “dry” storage, and storage period (24 hours, 3 days, or 7 days). The analysis excluded missing values, and the BH method was applied to adjust *p*-values for multiple testing.

Although TS from the same participant are not independent biological replicates, descriptive statistics were also used to summarize MTBC detection rates across specimen types, processing methods, and test platforms. Detection rates were calculated as proportions with corresponding percentages. Differences in MTBC detection across semi-quantitative categories of the Ultra sputum assay were similarly analysed.

## Results

### Phase one

Among participants with valid results for all tests, 5/38 (13%) had MTBC detected across all five TS by both qPCR and Ultra (Table 1). These five participants had “high” or “medium” semi-quantitative results on their sputum Ultra assay. In contrast, MTBC detection across the five TS was sporadic in the remaining participants. The one-way ANOVA analysis revealed that there were no statistically significant differences in Ct values among the five sequentially collected TS, as evidenced by an adjusted **p* *> 0.05, F(3) = 0,424, *p* = 0.736.

**Table 1:** Ultra semi-quantitative and qPCR results for tongue swabs from participants recruited during phase one.

PID	Ultra (sputum) semi-quantitative result	Ultra (TS 5)	Quantstudio (qPCR) results (storage temperature, storage duration)											
			TS 1 (2–8°C, 24 hours)			TS 3 (2–8°C, 24 hours)			TS 2 (−80°C, 1–5 months)			TS 4 (−80°C, 1–5 months)		
			HL-1	HL-2	VBB	HL-1	HL-2	VBB	HL-1	HL-2	VBB	HL-1	HL-2	VBB
1	high	low	positive	positive	positive	positive	positive	positive	positive	positive	positive	positive	positive	positive
2	high	vlow	positive	positive	positive	positive	positive	positive	positive	positive	positive	positive	positive	positive
3	high	error 5007	positive	positive	positive	positive	positive	positive	positive	positive	positive	positive	positive	positive
4	high	low	negative	negative	negative	positive	negative	negative	positive	positive	negative	positive	negative	negative
5	high	error 5007	positive	positive	positive	positive	positive	positive	positive	positive	positive	positive	negative	positive
6	high	low	positive	positive	positive	positive	positive	positive	positive	positive	positive	positive	positive	positive
7	high	medium	negative	negative	positive	negative	negative	positive	negative	negative	negative	negative	negative	negative
8	high	vlow	negative	negative	negative	negative	negative	positive	negative	negative	negative	negative	negative	negative
9	high	vlow	positive	positive	positive	positive	negative	positive	positive	positive	positive	positive	negative	positive
10	medium	low	positive	positive	positive	negative	positive	positive	positive	positive	positive	positive	negative	negative
11	medium	trace	negative	negative	negative	negative	negative	negative	negative	negative	negative	negative	negative	negative
12	medium	vlow	negative	negative	positive	negative	negative	negative	negative	negative	negative	negative	negative	positive
13	medium	error 5007	positive	positive	positive	negative	negative	negative	positive	negative	positive	positive	negative	positive
14	medium	not detected	negative	negative	negative	negative	negative	negative	negative	negative	negative	negative	negative	negative
15	medium	vlow	negative	invalid	positive	positive	negative	negative	positive	positive	positive	positive	positive	positive
16	medium	trace	positive	positive	positive	positive	positive	positive	positive	positive	positive	positive	positive	positive
17	medium	trace	positive	positive	positive	positive	positive	positive	positive	positive	positive	positive	positive	positive
18	medium	error 5007	positive	positive	positive	positive	positive	positive	positive	positive	positive	positive	positive	positive
19	medium	vlow	negative	negative	negative	positive	positive	positive	negative	negative	positive	negative	negative	negative
20	medium	not detected	negative	negative	negative	negative	negative	negative	positive	negative	positive	negative	negative	positive
21	medium	low	negative	negative	negative	negative	negative	negative	negative	positive	positive	negative	negative	positive
22	low	not detected	negative	negative	negative	negative	negative	negative	negative	negative	negative	negative	negative	negative
23	low	not detected	negative	negative	negative	negative	positive	positive	negative	negative	positive	negative	negative	positive
24	low	not detected	negative	negative	negative	negative	negative	negative	negative	negative	negative	negative	negative	negative
25	low	not detected	negative	negative	positive	negative	positive	negative	negative	negative	positive	negative	negative	negative
26	low	not detected	negative	negative	negative	negative	negative	negative	negative	negative	negative	negative	negative	negative
27	low	trace	positive	positive	positive	positive	positive	positive	positive	positive	positive	positive	positive	positive
28	low	not detected	negative	negative	negative	negative	negative	negative	negative	negative	negative	negative	negative	negative
29	low	trace	negative	negative	negative	negative	negative	negative	negative	negative	negative	positive	negative	negative
30	low	not detected	positive	negative	negative	negative	negative	positive	negative	negative	negative	negative	negative	negative
31	low	not detected	negative	negative	negative	negative	negative	negative	negative	negative	negative	negative	negative	negative
32	vlow	vlow	negative	negative	negative	negative	negative	negative	negative	negative	negative	negative	negative	negative
33	vlow	not detected	negative	negative	negative	negative	negative	negative	negative	negative	negative	negative	negative	negative
34	vlow	error 5007	negative	negative	positive	negative	negative	negative	negative	negative	negative	negative	positive	negative
35	trace	not detected	negative	positive	positive	negative	negative	positive	negative	positive	positive	negative	negative	negative
36	trace	not detected	negative	negative	negative	negative	negative	negative	negative	negative	negative	negative	negative	negative
37	trace	not detected	negative	negative	negative	negative	negative	positive	negative	negative	negative	negative	negative	negative
38	trace	not detected	negative	negative	negative	negative	negative	negative	negative	negative	negative	negative	negative	negative
**Total *n*/*N* (%)**		**18/38 (47)**	**13/38 (34)**	**13/38 (34)**	**18/38 (47)**	**13/38 (34)**	**13/38 (34)**	**18/38 (47)**	**15/38 (40)**	**15/38 (40)**	**19/38 (50)**	**15/38 (40)**	**10/38 (26)**	**16/38 (42)**

HL-1, initial heat lysis; HL-2, repeat heat lysis; VBB, vortex bead beating; vlow, very low; quantitative polymerase chain reaction; error 5007 indicates insufficient sample processing or a failure in the assay’s internal quality controls, positive indicates a MTBC-detected result by qPCR, negative indicates a MTBC not detected result by qPCR; invalid indicates that the assay control failed to amplify, Ultra refers to the Xpert MTB/RIF Ultra assay; *n*, refers to the number of TS where MTBC was detected; *N*, refers to the total number of TS tested.

When comparing TS processed with HL-only, MTBC was detected by qPCR in 26/76 (34%) of TS tested within 24 hours of collection and 30/76 (39%) of TS stored at –80°C. Mean Ct values for IS*6110* and IS*1081* did not differ significantly between fresh and frozen specimens (*t* (85) = −0.031, *p* = 0.98; 95% CI: –0.44 to 0.43).

**Fig 3:**
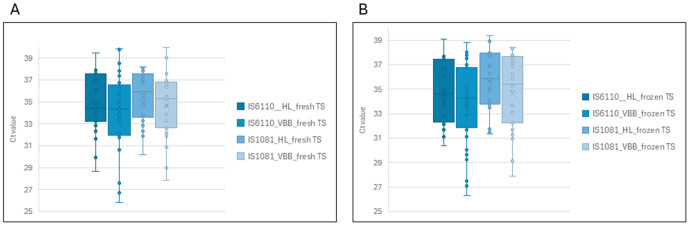
Ct values for insertion elements (*IS*6110 and *IS*1081) obtained on qPCR between tongue specimens that were heat lysed only compared with a combination of heat lysis and vortex beat beating. A represents TS that were processed “fresh” or within 24 hours of collection and B represents TS that were processed after freezing.

MTBC was detected in 51/152 (41%) specimens processed using HL-only and 71/152 (47%) after VBB. According to the paired *t-*test, there is a notable difference in mean Ct values following VBB between HL-only and VBB (*t* (85) = −9.663, *p* = (t (85) = −9.67, *p* = 2.422 x 10^-15^; 95% CI: −2.44 to −1.61). VBB generally produced Ct value ~0.08–1.62 lower than those from HL-only. Ct values for heat-lysed specimens remained consistent before and after storage (S1 Table), whereas VBB treatment led to a 1–4 cycle reduction in Ct values (Fig 3). Repeat HL did not improve sensitivity, as detection rates remained similar.

For Ultra TS testing, MTBC detection varied across the Ultra sputum semi-quantitative range with 18/38 (47%) testing positive for MTBC. A total of 5/38 (13%) Ultra TS specimens produced unsuccessful results.

### Phase two

The one-way ANOVA analysis revealed that there were no statistically significant differences in Ct values among sequentially collected TS, as evidenced by an adjusted **p* *> 0.05, F(4) = 1.315, *p* = 0.266. This suggests that swab collection order did not influence the measured Ct values within participants.

Among participants with valid results for all tests, 3/39 (8%) had all seven TS test positive for MTBC by qPCR (Table 2). Detection rates varied by storage condition. When stored “dry” at –80°C, MTBC was detected in 10/20 (50%) TS after 3 days and in 11/20 (55%) TS after 7 days. For TS stored at –80°C in TE buffer, MTBC was detected in 8/19 (42%) after 3 days and in 10/19 (53%) after 7 days. At 37°C, MTBC was detected in 10/19 (53%) TS after 3 days of “dry” storage, compared to 7/19 (37%) after 7 days. When stored in TE buffer at 37°C, MTBC was detected in 13/20 (65%) TS after 3 days and in 11/20 (55%) after 7 days.

**Table 2:** Ultra semi-quantitative and qPCR results for tongue swabs from participants recruited during phase two.

Participant number	Ultra semiquantitative result (sputum)	Ultra semiquantitative result (TS) MTM only	Ultra semiquantitative result (TS) MTM and SR	Quantstudio (qPCR) results (Storage temperature, transport condition, storage duration)								
				2-8°C, dry, 24 hours	−80°C, dry, 3 days	−80°C, dry, 7 days	−80°C, TE buffer, 3 days	−80°C, TE buffer, 7 days	37°C, dry, 3 days	37°C, dry, 7 days	37°C, TE buffer, 3 days	37°C, TE buffer, 7 days
1	high	medium	low	positive	positive	positive					positive	positive
2	high	trace	vlow	positive	positive	positive					positive	positive
3	high	error 2008	low	positive	negative	positive					positive	positive
4	high	low	low	negative	positive	positive					positive	positive
5	high	low	low	positive			positive	positive	positive	positive		
6	high	low	low	positive			positive	positive	positive	invalid		
7	high	trace	not detected	negative	positive	negative					positive	positive
8	high	low	vlow	negative	positive	positive					positive	positive
9	high	not detected	trace	negative			positive	positive	positive	positive		
10	high	low	vlow	negative	negative	positive				negative	positive	positive
11	high	low	trace	negative			positive	positive	positive	positive		
12	high	low	low	negative			positive	positive	positive	positive		
13	high	error 2008	medium	positive	positive	positive				negative	positive	invalid
14	high	high	low	negative			positive	positive	positive	invalid		
15	high	trace	not detected	negative			negative	negative	positive	positive		
16	medium	vlow	trace	positive			positive	positive	positive	positive		
17	medium	error 2008	low	positive	positive	positive				negative	positive	positive
18	medium	vlow	trace	positive	positive	positive					positive	positive
19	medium	vlow	vlow	negative			positive	negative	positive	positive		
20	medium	vlow	vlow	positive			negative	positive	negative	negative		
21	medium	not detected	not detected	negative	negative	negative					positive	negative
22	medium	vlow	not detected	positive			negative	negative	negative	negative		
23	medium	not detected	not detected	positive	negative	negative					negative	negative
24	medium	not detected	not detected	negative			negative	negative	negative	negative		
25	low	not detected	not detected	negative			negative	negative	negative	negative		
26	low	not detected	not detected	positive	negative	negative					negative	negative
27	low	vlow	not detected	negative			negative	positive	positive	negative		
28	low	not detected	not detected	positive			negative	negative	negative	negative		
29	low	error 2008	not detected	positive			negative	negative	negative	negative		
30	low	error 2008	not detected	positive			negative	negative	negative	negative		
31	low	not detected	not detected	positive			negative	negative	negative	negative		
32	low	not detected	not detected	negative	negative	negative						negative
33	low	not detected	not detected	negative	positive	positive					negative	negative
34	vlow	not detected	not detected	negative			negative	positive	negative	negative		
35	vlow	not detected	not detected	positive	positive	positive					positive	positive
36	trace	not detected	not detected	negative	negative	negative					negative	negative
37	trace	not detected	trace	negative	negative	negative					negative	negative
38	trace	not detected	not detected	negative	negative	negative					negative	negative
39	trace	not detected	trace	negative	negative	negative					positive	positive
**Total *n*/*N* (%)**		**18/39 (46%)**	**20/39 (51%)**	**18/39 (46%)**	**10/20 (50%)**	**11/20 (55%)**	**8/19** **(42%)**	**10/19 (53%)**	**10/19 (53%)**	**7/19 (37%)**	**13/20 (65%)**	**11/20 (55%)**

HL, heat lysis; VBB, vortex bead beating; vlow, very low; qPCR, quantitative polymerase chain reaction; MTM, molecular transport medium; error 2008 indicates a pressure related issue, positive indicates a MTBC-positive result by qPCR, negative indicates a MTBC not detected result by qPCR; Ultra refers to the Xpert MTB/RIF Ultra assay; invalid indicates that the assay control failed to amplify, cells without data indicate that testing was not performed under those storage conditions; *n*, refers to the number of TS where MTBC was detected; *N*, refers to the total number of TS tested.

**Fig 4:**
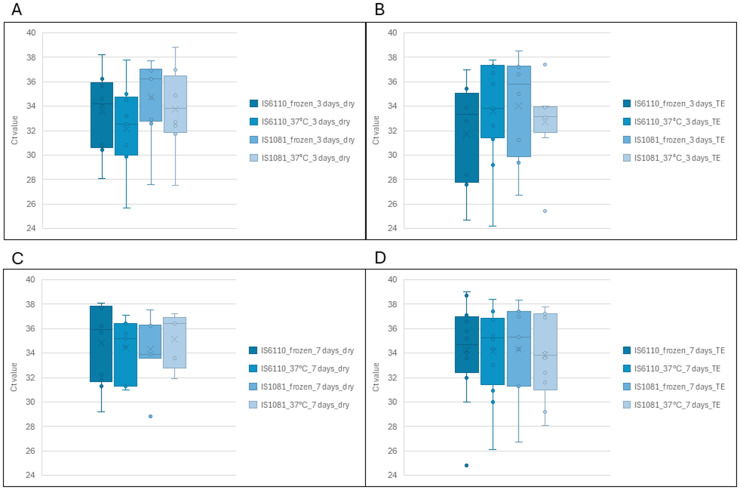
Comparison of Ct values for insertion elements (*IS*6110 and *IS*1081) obtained on qPCR between tongue swab specimens stored at different temperatures and durations. A represents TS stored “dry” either frozen or at 37°C for 3 days; B represents TS stored in TE buffer either frozen or at 37°C for 3 days; C represents TS stored “dry” either frozen or at 37°C for 7 days and D represents TS stored in TE buffer either frozen or at 37°C for 7 days.

Ct value comparisons between storage conditions showed that TS stored in TE buffer for 3 days had slightly lower Ct values, though with greater variability, compared to TS stored “dry” (Fig 4). After 7 days of frozen storage, Ct values were comparable between TS stored “dry” or in TE buffer. Similarly, TS stored in TE buffer at 37°C also showed lower Ct values with increased variability. Overall, while storage condition had some impact on both Ct value and consistency, the mean Ct values across groups varied only slightly, ranging from 32.48–34.28. Raw data are available in Supplementary S2 Table. Three-way ANOVA was performed to assess the impact of storage temperature, “dry” or TE buffer storage and storage period. The results revealed no statistically significant main effects of storage temperature, F (2,170) = 0.99, *p* = 0.374 or “dry” or TE buffer storage F (1,170) = 0.30, *p* = 0.583. In addition, the effect of storage period showed no statistical significance, F (1, 170) = 2.86, *p* = 0.093. None of the interaction terms reached statistical significance, there were no notable interactions between the storage temperature and buffer (F(1,170) = 0.013, *p* = 0.9087), storage temperature and storage period (F(1,170) = 0.159, *p* = 0.6910), buffer and storage period (F(1,170) = 0.032, *p* = 0.8587), or in the three-way interaction (F(1,170) = 0.014, *p* = 0.9054).

### Ultra results

Swabs processed using MTM alone demonstrated MTBC detection on 18/39 (46%) participants with 5/39 (13%) specimens producing errors. When using a combination of MTM and SR buffer, MTBC was detected in 20/39 (51%) participants without any errors.

## Discussion

In this study, we assessed whether different transport and storage conditions affect the performance of TS for detecting MTBC. In addition, we also modified processing methods in the laboratory to determine if assay sensitivity could be improved.

Phase 1 of the study showed that MTBC could be detected from five serially collected TS per participant, with a 1–2 Ct value variation between TS; however, this difference was not statistically significant. Results also demonstrated that MTBC detection can be sporadic, especially on those participants with a lower bacillary load, as determined by the Ultra sputum semi-quantitative result. Storage of TS at −80°C for up to 5 months, showed similar detection of MTBC, compared to testing within 24 hours after 2–8°C storage. The results of the paired *t*-test comparison suggest that the storage condition did not significantly influence IS*6110* and IS*1081* Ct values. The addition of VBB improved detection of MTBC and produced lower Ct values compared to HL alone. This is not surprising, given the lipid-rich cell wall of *Mycobacterium tuberculosis*, which is likely more resistant to disruption by heat alone. VBB facilitates mechanical disruption using beads to physically break open cells, thereby potentially facilitating DNA release, resulting in lower Ct values. Bead beating has been shown to improve recovery of other organisms compared to vortexing alone [10]. Phase 1 also demonstrated that TS were compatible with the Ultra assay, but we believed that assay sensitivity could be improved using protocol modification.

In Phase 2, TS were pre-processed using a combination of HL and VBB prior to qPCR testing. Although the sample size and storage duration (up to 7 days) were limited, MTBC detection was comparable between TS stored “dry” and those stored in TE buffer at –80°C. While some variation was noted, such as higher detection after 3 days at 37°C and lower Ct values for TS stored in TE buffer at –80°C, these differences did not reach statistical significance. Similarly, after 7 days, MTBC detection was somewhat higher in “dry” TS stored at –80°C compared with those stored in TE buffer, although Ct values remained similar across all conditions. Overall, our findings indicate that MTBC detection and Ct values were not significantly affected by “dry” or buffer storage, storage temperature, or storage duration within the conditions evaluated.A storage duration beyond 7 days was not evaluated, as this typically exceeds the time specimens take to reach laboratories in South Africa [11]. Additionally, prolonged storage at room temperature or higher, especially for respiratory specimens, increases the risk of overgrowth by contaminants, compromising downstream testing [12].

When using SR buffer alone, the TS Ultra error rate was high, indicating a specimen processing issue, however, MTBC was detected in 45% of participants, showing TS compatibility with the assay. MTM has been shown to improve MTBC detection in sputum with low bacillary loads while also stabilizing specimens for transport [13]. Swabs, for Ultra testing, were therefore transported in MTM in phase 2. MTBC was detected in 46% of TS transported in MTM only with an error rate of 13% while detection improved to 51%, with no errors produced, when a combination of MTM and SR buffer was used suggesting that our hypothesis was correct in that SR buffer may be required to for processing of TS prior to testing on Ultra. Although the buffer combination with MTM improved detection, it was subsequently found to be difficult to procure MTM in SA. There was also the consideration of the increased cost associated with purchasing MTM compared to transporting a TS without any buffer.

While this was not a comprehensive study and definitive conclusions cannot be drawn, study findings suggest that TS can be transported “dry” or in TE buffer at temperatures up to 37°C without compromising sample integrity. These results provided valuable insights that informed the design of subsequent, larger clinical performance evaluations conducted by our group to further optimize TS testing for MTBC detection.

Despite these promising results, study limitations must be acknowledged. The small sample size and evaluation of storage duration for only up to 7 days limit conclusions on longer-term stability. MTBC detection also varied between participants, particularly those with low bacillary loads. Future studies with larger sample sizes are recommended to ensure adequate statistical power. Although MTM improved Ultra detection, its high cost and limited availability in South Africa may restrict its use in programmatic settings.

## Conclusions

This study demonstrates that the diagnostic performance of TS are influenced by pre-processing methods. Key optimizations, such as VBB and use of diluted SR buffer can improve MTBC detection. These findings support the continued development and implementation of TS-based TB diagnostics, particularly in settings where sputum collection is challenging.

## Supporting information

S1 Table Raw qPCR results and Xpert MTB/RIF Ultra results from phase one. Comparison of *Mycobacterium tuberculosis* complex (MTBC) detection using Xpert MTB/RIF Ultra and qPCR from tongue swabs under different storage and processing conditions. Each row represents an individual participant (PID). Swabs were either tested within 24 hours or after storage at −80°C. Results from Xpert Ultra testing of sputum and TS 5 are shown. qPCR testing targeted IS*6110* and IS*1081* insertion sequences under two storage conditions (2–8°C for 24 hours and −80°C for 1–5 months), across three processing methods: Heat Lysis 1 (HL-1), Heat Lysis 2 (HL-2), and vortex bead beating (VBB).
(XLSX)

S2 Table Raw qPCR results and Xpert MTB/RIF Ultra results from phase two. Assay results from paired sputum and TS specimens tested using Xpert Ultra and qPCR, under various transport and processing conditions. Each row represents an individual participant (PID). Xpert Ultra testing was performed on sputum and TS stored in PrimeStore® Molecular Transport Medium (MTM) alone or combined with Sample Reagent (SR). Detection of *Mycobacterium tuberculosis* complex (MTBC), semiquantitative grading, cycle threshold (Ct) values for IS, and presence of rifampicin (RIF) resistance-associated mutations (rpoB1–rpoB4) are reported. qPCR testing targeted IS*6110* and IS*1081* on freshly collected TS (<24 hours), frozen TS stored at −80°C, and TS stored at 37°C. Swab number identifiers are provided for traceability between conditions and assays.
(XLSX)
